# Phenotypic diagnosis and genotypic identification of *Bacillus cereus* causing subclinical mastitis in cows

**DOI:** 10.14202/vetworld.2023.888-894

**Published:** 2023-05-07

**Authors:** Rasha H. Eid, Noha E. Aref, Eman S. Ibrahim

**Affiliations:** 1 Department of Mastitis and Neonatal Disease, Animal Reproduction Research Institute, Agriculture Research Center, Giza, Egypt; 2 Department of Microbiology and Immunology, National Research Centre, Giza, Egypt

**Keywords:** antibiotics, dairy cows, Egypt, lactation

## Abstract

**Background and Aims:**

Bovine mastitis is a disease that affects dairy cows and impacts the global dairy industry. *Bacillus* spp. can infect the mammary gland during lactation, intramammary treatment, or dry cow therapy. This study aimed to isolate and identify *Bacillus* spp. in raw milk samples from cows with subclinical mastitis from dairy farms in Beheira, Giza, Alexandria, and Menoufia Governorate, Egypt. We also investigated their antibiotic sensitivity and detected the enterotoxigenic and antibiotic resistance genes.

**Materials and Methods:**

A total of 262 milk samples (15-20 ml each) were examined microscopically, biochemically, and phenotypically. A polymerase chain reaction was used for genotypic identification and detecting antibiotic-resistance and enterotoxigenic genes. Antibiotic sensitivity was tested using the agar well diffusion test.

**Results:**

*Bacillus cereu*s was identified in 47.7% of samples. *Nhe* and *hblD* enterotoxin genes were found in 93.64% (103/110) and 91.82% (101/110) of the samples, respectively. Tetracycline and β-lactam antibiotic-resistance genes were present in 0% (0/110) and 98.18% (108/110), respectively, of the samples. All isolates were resistant to cefepime, cefixime, and oxacillin, while they were susceptible to amoxicillin-clavulanic, chloramphenicol, ampicillin/sulbactam, and levofloxacin.

**Conclusion:**

These results highlight the need to promote awareness regarding *B. cereus*, the most common pathogen causing mastitis in Egyptian dairy cows. We also emphasized that antibiotic misuse during mastitis is a potential public health threat.

## Introduction

Bovine mastitis is a disease affecting dairy cows characterized by pathological, chemical, and physical changes in the milk-producing glandular tissues [1]. It is a pernicious disease of great concern for the global dairy industry, leading to decreased milk production and rejected milk [2]. *Bacillus cereus* is a rod-shaped, Gram-positive, facultative-anaerobic, and endospore-forming pathogen that causes mastitis in cows and severe food poisoning in humans [3]. The spores can survive in dry and hot conditions and stay dormant for several years. They are resistant to heat and chemicals [4]. Soil, straw, and other fodders are the most common contaminants in dairy farms. The bedding used is also a potential contaminant when the cows are housed indoors during winter. Contaminated udders eventually result in the presence of *B. cereus* in raw milk [5]. *Bacillus* spp. can also infect the mammary gland during lactation, intramammary treatment, or dry cow therapy. Moreover, it can be introduced into the mammary gland through unsterilized injections. Some *Bacillus* spp. can cause fatal gangrenous mastitis. *Bacillus cereus* is the most common foodborne bacteria in raw milk and dairy farm environments [6]. *Bacillus cereus* is a species complex with high phenotypic and genotypic similarity [7]. The proteins encoded by the *groEL* and *sodA* genes are essential for bacterial cell viability, and hence, these genes can be used for phylogenetic analysis to identify *B. cereus*. The *groEL* gene has been investigated as a phylogenetic marker [8]. However, genomic studies are required to assess the genetic mechanisms and factors enabling toxin production to differentiate between *Bacillus* spp. [9].

*Bacillus cereus* causes several diseases in humans and animals [10]. They are the most frequently isolated foodborne bacterial pathogens and can produce several powerful toxins [11]. Consequently, they endanger public health by forming spoilage enzymes and toxins in dairy products, resulting in enormous economic losses [9]. *Bacillus cereus* causes two types of food poisoning: diarrheal and emetic, which negatively affect human health. The diarrheal type is linked to the production of enterotoxins such as *hemolysin BL* (*hbl*) and *non-hemolytic enterotoxin* (*nhe*) [12]. Further, tetracycline-resistant genes *tetA* and *tetB* have been reported for the 1^st^ time in *B. cereus* [13]. Most *B. cereus* strains are resistant to β-lactam antibiotics as they produce the lactamase enzyme [14]. *Bacillus cereus* infections are still primarily treated using antibiotics. However, the emergence of antibiotic-resistant *B. cereus* strains due to antibiotic misuse [15] and transmission of resistance genes through horizontal gene transfer [16] has resulted in the failure of antibiotic treatments.

Therefore, understanding the antibiotic resistance profile is crucial before treating *B. cereus*. Further, the importance of *B. cereus* as a major cause of mastitis among Egyptian dairy farms should be elucidated. This study aimed to detect antibiotic-resistance and toxigenic genes from *B. cereus* found in raw milk of sub-mastitic cows from different governorates in Egypt.

## Materials and Methods

### Ethical approval

Ethical approval was not required for this study; however, samples were collected as per the standard sample collection procedure.

### Study period and location

The study was conducted from January 2018 to January 2020 at the National Research Centre in Dokki, Egypt and Animal Reproduction Research Institute Agriculture Research Center (ARC), Giza, Egypt.

### Sample collection

A total of 262 milk samples were collected aseptically using sterile vials from cows with subclinical mastitis from dairy farms in Beheira, Giza, Alexandria, and Menoufia governorates, which were suffering from decreased milk yield, recurrent mastitis, and failure of antibiotic treatment. The milk samples were placed immediately in an ice container and transported to the microbiology laboratory. The samples were collected in compliance with the rules of the local Commission for Ethics in Animal Experimentation and Investigation

### Isolation and identification of *B. cereus* strains

#### Bacterial culture

The milk samples were cultured on Bacillus selective agar (HiMedia, India), and after 24 h–48 h of incubation at 37°C, the plates were examined for bacterial growth. The *B. cereus* colonies displayed a distinct turquoise-peacock blue color and were surrounded with egg yolk-like precipitate of the same diameter. The color of the indicator dye around the colony remained unchanged as *B. cereus* does not ferment mannitol. We performed morphological and biochemical tests on all suspected *B. cereus* colonies. The Gram-stained smears were microscopically examined to identify their cell shape, motility, and hemolysis. We also evaluated nitrate reduction and the production of enzymes, including catalase, oxidase, urease, and lecithinase [17].

#### Identification of *B. cereus* using HiCrome^™^ Bacillus agar (HiMedia)

We observed one or more blue colonies on each Bacillus selective agar media plate. The lecithin-positive colonies appeared as light-blue colored, large, flat colonies with blue centers, and pink edges on chromogenic *B. cereus* agar after adding *Bacillus* Selective Supplement (FD324) and incubating at 30°C for 24–48 h [18].

### Genotypic characterization of *B. cereus* and associated virulence genes

#### Detection of the groEL gene

A polymerase chain reaction (PCR) analysis was performed on all 125 chromogenic-positive isolates. A single typical colony was inoculated on brain heart infusion broth and incubated overnight at 37°C. We investigated the potential of the *groEL* gene as a phylogenetic marker by extracting deoxyribonucleic acid (DNA) from the broth culture using a positive reference strain (*B. cereus* ATCC 14579).

#### Detection of the virulence genes (enterotoxigenic and antibiotic resistance genes)

A polymerase chain reaction was performed to detect the virulence genes, including *hblD* and *nhe*, *tetA*, and beta lactam-resistant (*bla*) genes in the positive isolates identified using *groEL*.

### Deoxyribonucleic acid extraction

The DNA was extracted from the samples using the QIA amp DNA Mini kit (Qiagen, Germany, GmbH) based on the manufacturer’s recommendations with slight modifications.

### Oligonucleotide primers

The PCR primers, supplied by Metabion (Germany), are listed in Table-1 [19–23].

**Table-1:** Primers sequences, target genes, amplicon sizes and cycling conditions.

Target gene	Primer sequences	Amplified segment (bp)	Primary denaturation	Amplification (35 cycles)			Final extension	Reference

				Secondary denaturation	Annealing	Extension		
*Bacillus cereus groEL*	TGCAACTGTATTA GCACAAGCT	533	94°C 5 min	94°C 30 s	55°C 40 s	72°C 45 s	72°C 10 min	[19]
	TACCACGAAGTTT GTTCACTACT							
*Nhe*	AAG CIGCTCTT CGIATTC	766	94°C 5 min	94°C 30 sec	49°C 40 s	72°C 45 s	72°C 10 min	[20]
	ITI GTT GAA ATA AGC TGT GG							
*hblD*	AGT TAT TGC AGC TAT TGG AGG	148	94°C 5 min	94°C 30 s	56°C 30 s	72°C 30 s	72°C 7 min	[21]
	GTC CAT ATG CTT AGA TGC TGT GA							
*tetA*	GGCGGTCTTCT TCA TCA TGC	502	94°C 5 min	94°C 30 s	58°C 40 s	72°C 45 s	72°C 10 min	[22]
	CGGCAGGCAGA GCA AGT AGA							
*Blab*	CATTGCAAGTTG AAG CG AAA	680	94°C 5 min	94°C 30 s	50°C 40 s	72°C 45 s	72°C 10 min	[23]
	TGTCCCGTAA CTTCCAGCTC							

*hbl=hemolysin BL, nhe=Non-hemolytic enterotoxin, tetA=*tetracycline-resistant gene *A, Blab=Beta lactam-resistant b*

### Polymerase chain reaction amplification

The PCR reaction was performed using a reaction mixture containing 12.5 μL Emerald Amp Max PCR Master Mix (Takara, Japan), 1 μL of each primer (20 pmoL), 5.5 μL water, and 5 μL DNA template in a final volume of 25 μL using an Applied Biosystems thermal cycler type 2720.

### Analysis of PCR products

The PCR products were separated by running a 1.5% agarose gel (Applichem, Germany) at a 5 V/cm gradient in 1× Tris borate ethylenediaminetetraacetic acid buffer at room temperature. Each lane was loaded with 15 μL of the product, and the fragment sizes were determined using the Generuler 100 bp ladder (Fermentas, Germany). The gel was photographed using a gel documentation system (Alpha Innotech, Biometra, Germany), and the data were analyzed using computer software.

### Antibiotic sensitivity test

The antibiotic susceptibility was tested using 15 disks (Oxoid, UK) containing vancomycin (VA, 30 μg), amoxicillin-clavulanic (30 μg), chloramphenicol (C, 30 μg), cefuroxime (CXM, 30 μg), ampicillin/sulbactam (10 μg), cefepime (FEP, 30 μg), and ciprofloxacin (CIP, 5 μg). After that, single colonies were selected and suspended in 0.85% physiological saline, adjusted to 0.5 McFarland standards, and distributed on a Mueller-Hinton agar plate. After drying, the inoculum antibiotic disks (HiMedia) were deposited on the plate’s surface and incubated overnight at 37°C. The strain was classified as susceptible (S) or resistant (R) based on the inhibition zone’s diameter [24].

### Statistical analysis

Data presented in tables as percentages were subjected to an exact test using IBM-SPSS 20.0 software (IBM Corp., NY, USA). In addition to Pearson Chi-square, ‘Fisher’s Exact, Linear-by-Linear Association, and McNemar tests were also performed.

## Results and Discussion

*Bacillus cereus* is a Gram-positive bacteria found in nature [25]. When present in milk, *B. cereus* causes milk spoiling, which results in food poisoning in humans [26]. It is considered one of the major causes of mastitis in cows on dairy farms [27].

In the present study, we isolated 125 (93.2%) *B. cereus* strains from cows with subclinical mastitis based on colony morphology and biochemical tests (Table-2). These isolates were confirmed by culturing on chromogenic *B. cereus* agar media (Table-3), consistent with the study by Hammad *et al*. [28] reporting that *B. cereus* is 85% prevalent in raw milk in Egypt. However, this prevalence rate is higher than others reported by Meng *et al*. [6], Hassan *et al*. [29], Haughton *et al*. [30], Rezende-Lago *et al*. [31], who found that *B. cereus* were 46.6%, 59%, 50%, and 61.1% prevalent, respectively. Conversely, other studies by Alemneh [32], Seblewongel [33], and Gilles *et al*. [34] found lower isolation rates of 15.4%, 15.86%, and 15.4%, respectively. Furthermore, Hayat *et al*. [35] determined that *B. cereus* is associated with subclinical mastitis in buffaloes in swats, with a (3.27%) prevalence. In addition, Ghazali *et al*. [36] identified that 23 of 78 milk samples from subclinical mastitic goats contained *B. cereus*. These variations in results can be attributed to weather variations or the hygiene conditions in the farms that differ from those observed in this study.

**Table-2:** Samples from cow milk cultured on *Bacillus* selective agar media.

Total number of individual cow milk samples	Cultured on *Bacillus* selective agar			

	Positive No.		Negative No.	
	
	No.	%	No.	%
262	134	51.14*	128	48.85*

* Non-significant

**Table-3:** *Bacillus cereus* isolates confirmed by chrome agar media.

Total no. of individual cow milk samples	Cultured on *Bacillus* selective agar			

	Positive No		Negative No	
	
	No.	%	No.	%
262	134	51.14*	128	48.85*

* Non-significant. However, the percentages of positive isolates by *Bacillus* selective agar media and chrome agar media are significantly low (p = 0.014) and the negative isolates are high (p = 0.014) compared to those confirmed by PCR using *groEL* gene (Figure-3)

**Figure-1:**
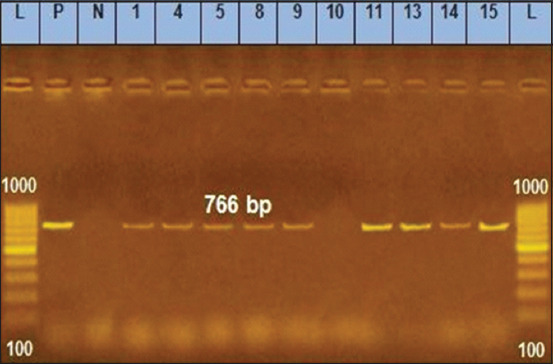
*Non-hemolytic enterotoxin* gene at 766 bp; lane L: 100 bp ladder, lane P: Positive Control, Lane N: Negative control, Lanes 1–15 representative to *Bacillus cereus* isolates.

**Figure-2:**
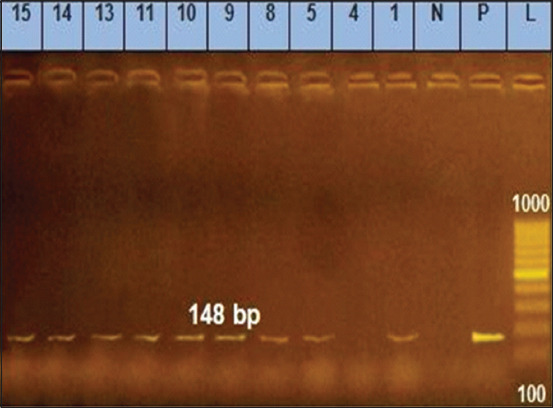
*Hemolysin BL D* gene at 148 bp; lane L: 100 bp ladder, lane P: Positive control, Lane N: Negative control, Lanes 1–15 representative to *Bacillus cereus* isolates.

Polymerase chain reaction analysis is a simple, fast, and reliable tool for effectively identifying microorganisms from numerous sources [37]. The groEL gene was used to detect B. cereus [38], as its efficacy has been demonstrated in previous phylogenetic research [39]. In this study, the PCR results revealed that 110 isolates (88%) harbored the *groEL* gene while 15 isolates (22%) did not (Table-4). The toxin hemolysin is a virulence factor that can potentially cause diarrhea and necrosis [40]. Species containing the enterotoxin genes *nhe* and *hbl* primarily cause food deterioration, resulting in food poisoning [41]. Bacteria produce diarrheal toxins when they multiply in the intestines. At least three bacterial toxins are known to be involved in diarrheal syndrome: *hbl*, *nhe* [42], and the genes *hblA*, *hblC*, and *hblD* that encode the three-component *hemolysin BL enterotoxin* [43]. In this study, the toxigenic genes (*nhe and hblD*) were detected in 110 *B. cereus* isolates, of which 103 were *nhe*-positive (93.64%) and 101 were *hblD*-positive (91.82%) (Table-5 and Figures-1 and 2) [44]. Remarkably, *nhe* was identified in all isolates, while only 50.7% had *hbl* genes. However, Owusu-Kwarteng *et al*. [7] found that 13% (12/96) of the isolates found in the raw milk and other dairy products of farm-raised cattle had all three hemolytic *hbl* complex enterotoxin genes (*hblA*, *hblC*, and *hblD*), whereas 25% had no *hbl* gene, and 63% had one or more of the three *hbl* genes. Moreover, they showed that 14% (13/96) had only one *nhe* gene, 60% (57/96) had all three *nhe* genes (*nheA*, *nheB*, and *nheC*), and 8% had no *nhe* genes. In addition, Meng *et al*. [6] showed that 12.77% and 8.51% of *B. cereus* isolates obtained from farm environments and raw milk harbored the *hblACD* and *nheABC* genes, respectively. The high percentage of toxigenic genes indicates the importance of detecting virulence factors to understand the involvement of the production of various toxins and enzymes.

**Table-4:** *Bacillus cereus* isolates positive on chrome agar confirmed by PCR using *groEL* gene.

Total number of positive isolates on chrome agar confirmed by PCR	Positive number by PCR using *groEL* gene		Negative number by PCR using *groEL* gene	
	
	No.	%	No.	%
125	110	88*	15	12*

* None significant. The difference between the percentage of isolated *Bacillus cereus* on Bacillus selective agar media [Table-2], chrome agar media [Table-3] and confirmed by PCR using *groEL* gene [Table-4] are not significantly different compared to non-isolated ones. PCR: Polymerase chain reaction

**Table-5:** Virulence genes detected in *B. cerues* isolates.

Total number of *B. cereus* isolates confirmed by PCR	Virulence genes							

	Enterotoxin gene				*bla* gene		*tetA* gene	

	*hblD*		*nhe*					
			
	No.	%	No.	%	No.	%	No.	%
110	101	91.82*	103	93.64*	108	98.18*	0	0

* Significant at p *<* 0.001. The percentages of enterotoxin *hblD* and *nhe* genes and *bla* gene are higher (p < 0.001, p *=* 0.046) than *tetA* gene identified by PCR using Lambda and Somers’d exact tests [Table-3]. *B. cereus=Bacillus* *cereus*, PCR: Polymerase chain reaction, *hbl=hemolysin BL*, *nhe*=*Non-hemolytic enterotoxin*, *tetA*=tetracycline-resistant gene *A*, *Bla*=Beta lactam-resistant

**Figure-3:**
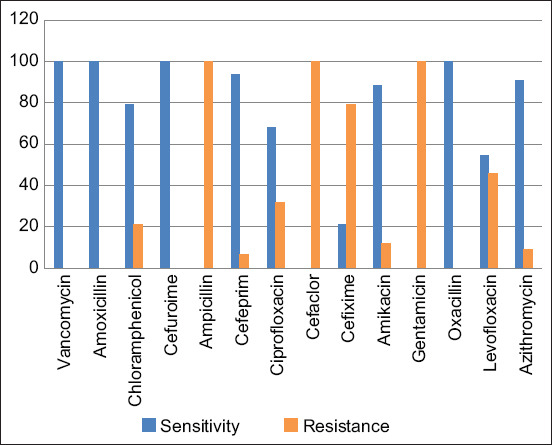
Percentages of antibiotic resistance and sensitivity for isolated bacteria.

*Bacillus cereus* is a global health threat as they are extremely resistant and have genetic mechanisms for responding to various environmental conditions. The antibiogram pattern against several commonly used antibiotics showed 100% resistance to FEP and cefixime (CFM). However, they are 100% sensitive to amoxicillin, C, ampicillin, and levofloxacin (LE), followed by CIP (93.6%), azithromycin (AZM) (90.9%), getamicin (88.2%), CXM (79.1%), VA and cefaclor (CF) (68.2%), tetracycline (TE) (54.5%), and amikacin (AK) (20.9%) (Table-6 and Figure-3). Our results were consistent with Owusu-Kwarteng *et al*. [7], who reported that *B. cereus* was susceptible to C (99%) and CIP (100%). The results by Sadashiv and Kaliwal *et al*. [45] showed that *B. cereus* was resistant to ampicillin (50.67%), C (6.33%), and AZM (5.42%). Furthermore, *B. cereus* showed 54.75%, 51.13%, 12.21%, 17.64%, and 7.69% resistance to CFM, CF, gentamicin (GEN), AK, and TE, respectively, which contradicted our findings. Moreover, similar results were detected regarding CIP (4.07%) and VA. According to Rosovitz *et al*. [46], *B. cereus* is susceptible to VA, and most strains are sensitive to C, CIP, erythromycin, and GEN. Few *B. cereus* strains are moderately sensitive to clindamycin and TE [47]. Tetracycline resistance was observed in 45.5% (50/110) of *B. cereus* isolates, significantly higher than that reported by Whong and Kwaga [48], who showed that 6.7% of *B. cereus* isolates were TE-resistant. These results indicate the importance of effectively selecting specific antibiotics to treat antibiotic-resistant *B. cereus* strains in dairy farms.

**Table-6:** The antibiotic sensitivity tests used for *Bacillus cereus* isolates.

Antibiotic discs	Sensitive		Resistance	
	
	No.	%	No.	%
VA 30 µg	75	68.2	35	31.8
AMC 30 µg	110	100	0	0
C 30 µg	110	100	0	0
CXM 30 µg	87	79.1	23	20.9
A/S 10 µg	110	100	0	0
FEP 30 µg	0	0	110	100
CIP 5 µg	103	93.6	7	6.4
CF 30 µg	75	68.2	35	31.8
CFM30 µg	0	0	110	100
AK 30 µg	23	20.9	87	79.1
GEN 10 µg	97	88.2	13	11.8
OX 1 µg	0	0	110	100
LE 5 µg	110	100	0	0
TE 30 µg	60	54.5	50	45.5
Azm 15 µg	100	90.9	10	9.1

VA=Vancomycin, AMC=Amoxicillin clavulanic, C=Chloramphenicol, CXM=Cefuroxime, A/S=Ampicillin/Sulbactam, FEP=Cefepime, CIP=Ciprofloxacin, CF=Cefaclor, CFM=Cefixime, AK=Amikacin, GEN=Gentamicin, OX=Oxacillin, LE=Levofloxacin, TE=Tetracycline, AZM=Azithromycin

Pearson Chi-square, Likelihood Ratio, Fisher’s Exact Test, and Linear-by-Linear Association (p < 0.0001), Goodman and Kruskal tau, and Somers’d (p < 0.0001) indicated a significant difference between resistance and sensitivity to different antibiotics (Table-6). All symmetric measures of the exact test (Phi, Cramer’s V, Contingency Coefficient, Kendall’s tau-b, Kendall’s tau-c, Gamma, Spearman Correlation, and Pearson’s R) showed significant (p < 0.0001) with fair (k = −0.153) and significant (p = 0.0001) measure of agreement (Kappa).

Based on these findings, suspected *B. cereus* infections should be clinically treated with VA or LE rather than broad-spectrum cephalosporins and penicillin. Furthermore, we found that several *B. cereus* isolates were multidrug-resistant, implying that raw milk infected with *B*. cereus is a major concern [49]. We agree with Chen *et al*. [50], who discovered that VA should be the drug of choice for *B. cereus* infections.

**Figure-4:**
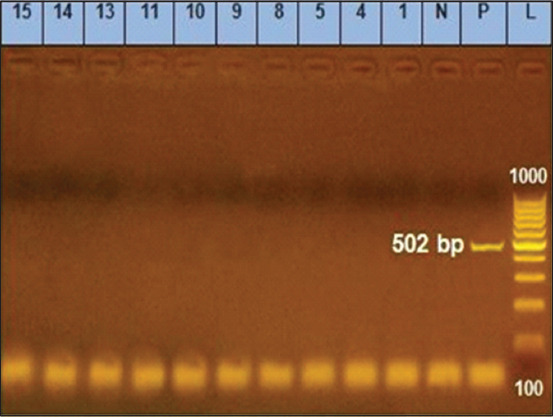
Tetracycline-resistant *A* gene at 502 bp; lane L: 100 bp ladder, lane P: Positive control, Lane N: Negative control, Lanes 1–15 representative to *Bacillus cereus* isolates.

The molecular examination of the antibiotic-resistant genes *bla* and *tetA* revealed that despite the absence of the *tetA* gene (Table-5 and Figure-4), 45.5% (50/110) of *B. cereus* isolates displayed TE resistance phenotypically. Our results agree with Agers *et al*. [51], who found that phenotypically three isolates showed TE resistance despite the lack of *tetA, tetB, or tetC*. This might be due to the presence of other TE resistance genes, for example, *tetM and tetL*, or other gene mutations. When a bacterial cell becomes resistant, it can swiftly transmit the antibiotic resistance genes to numerous species [52], transferring TE resistance genes [53].

**Figure-5:**
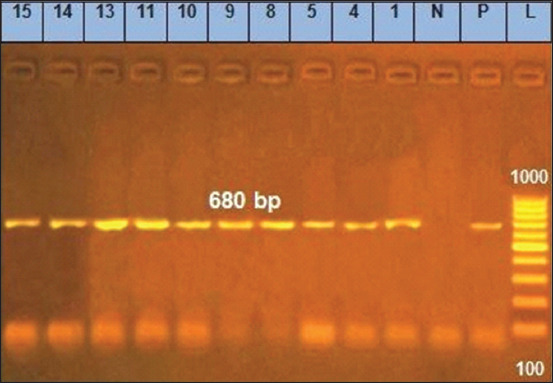
*Beta lactam-resistant* gene at 680 bp; lane L: 100 bp ladder, lane P: Positive control, Lane N: Negative control, Lanes 1–15 representative to *Bacillus cereus* isolates.

*Bacillus cereus* isolated from milk and dairy products were mostly resistant to β-lactam antibiotics. *Bacillus* species contain genes encoding β-lactamase [50], making most *B. cereus* isolates resistant to β-lactam antibiotics. Furthermore, it shows resistance to third-generation cephalosporins. Molecular examination showed that 98.18% (108/110) of the identified carried the *bla* gene (Table-5 and Figure-5), consistent with the results by Abd El-Tawab *et al*. [54], who detected the *bla* gene in all obtained isolates (100%).

## Conclusion

The *B. cereus* strains isolated from subclinical bovine mastitis cases showed high rates of resistance to most tested antibiotics due to the presence of several antibiotic-resistant and virulence genes (*hblD* and *nhe*). This suggested the emergence of multidrug resistance among these isolates in Egypt, which makes it necessary for milk producers and conventional dairy processors to follow strict sanitary and manufacturing practices to avoid contamination and subsequent disease outbreaks caused by *B. cereus*. Furthermore, it is crucial to determine the antibiotic resistance profile of *B. cereus* to identify treatment regimens and raise awareness for *B. cereus* as one of the most important causes of mastitis.

## Authors’ Contributions

All authors participated in the study design. RHE and NEA: Sample collection. RHE, NEA, and ESI: Isolation and identification of isolates. RHE, NEA, and ESI: Antibiogram profile, and molecular characterization of *B. cereus*. RHE and ESI: Molecular characterization of antibiotic-resistance genes and virulence genes. RHE and NEA: Data analysis. NEA and ESI: Drafted the manuscript. All authors have read, reviewed, and approved the final manuscript.
